# P-1763. Implementation of a Pharmacy-based Automated MRSA PCR Screening Intervention to Guide Discontinuation of Vancomycin for Pneumonia

**DOI:** 10.1093/ofid/ofae631.1926

**Published:** 2025-01-29

**Authors:** Reem Azem, Elie Saade, Courtney Veltri, Nicholas Newman, Natalie Kolehmainen, Khoi Dang, Lisa M Stempak, Brigid Wilson, Leila S Hojat

**Affiliations:** University Hospitals Cleveland Medical Center, Cleveland, Ohio; Case Western Reserve University, Cleveland, OH; University Hospitals Cleveland Medical Center, Cleveland, Ohio; University Hospitals Cleveland Medical Center, Cleveland, Ohio; University Hospitals Ahuja Medical Center, Beachwood, Ohio; University Hospitals Ahuja Medical Center, Beachwood, Ohio; University Hospitals, Lyndhurst, Ohio; VA Northeast Ohio Healthcare System, Cleveland, Ohio; Case Western Reserve University/ University Hospitals Cleveland Medical Center, Cleveland, OH

## Abstract

**Background:**

Vancomycin is administered for suspected methicillin-resistant *Staphylococcus aureus* (MRSA) pneumonia in hospital settings, but is also associated with adverse effects, emergence of resistance, and requires lab and pharmacy monitoring. MRSA nares screening has excellent negative predictive value for MRSA pneumonia and results faster than culture. We sought to decrease vancomycin overuse by identifying patients at low risk for MRSA pneumonia using rapid polymerase chain reaction (PCR) as part of an automated pharmacy-based intervention.

**Figure:**
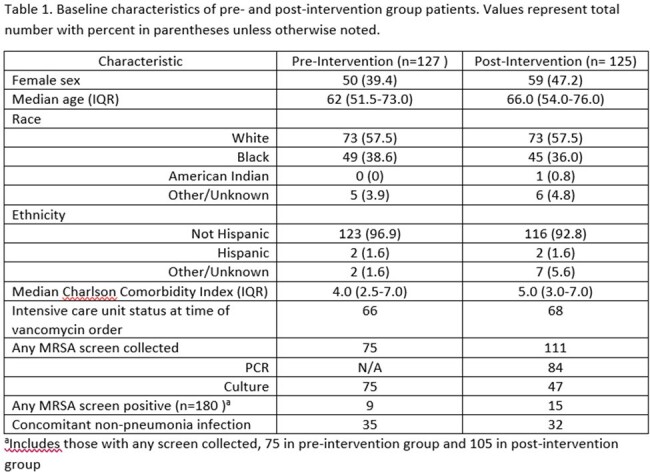

**Methods:**

We performed a retrospective cohort study of inpatients prescribed vancomycin for pneumonia at University Hospitals Cleveland Medical Center from November to May 2022 (pre-intervention) and November 2022 to May 2023 (post-intervention). In June 2022, we implemented a rapid MRSA PCR screen that was automatically added to orders when vancomycin was ordered for pneumonia. We compared median time to vancomycin discontinuation between the two groups. We performed a desirability of outcome ranking and response adjusted for duration of antibiotic risk (DOOR-RADAR) analysis, ranking patients according to duration of therapy within clinical categories to assess the effect of the intervention on patient-centered outcomes.

**Figure:**
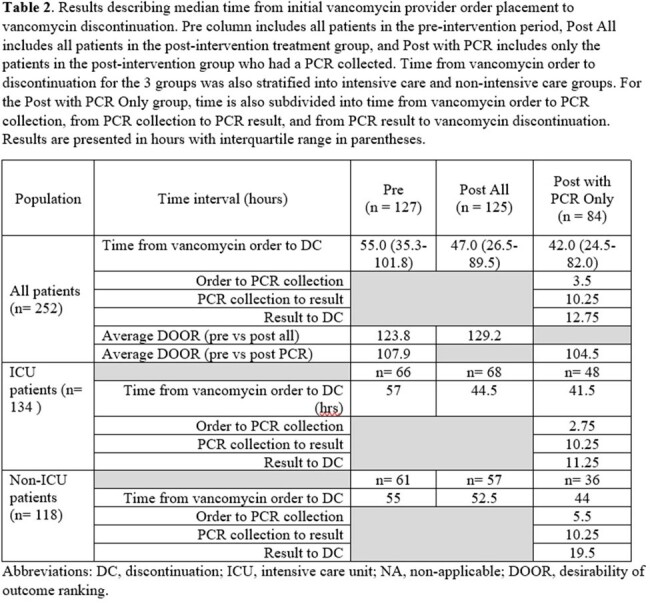

**Results:**

We identified 127 pre-intervention and 125 post-intervention patients (**Table 1**). Median vancomycin order to discontinue time was lower in post-intervention patients, though this was meaningfully different only for patients whose PCR test was collected after the automated order was placed (41.75 vs 55 hours respectively, p = 0.035) (**Table 2**). Most patients in the post-intervention group with vancomycin duration >48 hours had delayed collection or were treated for other infections (**Figure 1**). The DOOR ranks for median time to discontinuation within clinical outcome categories were not meaningfully different between the two groups (p >0.05) (**Table 2** and **Figure 2**) which may relate to sample size.

**Figure:**
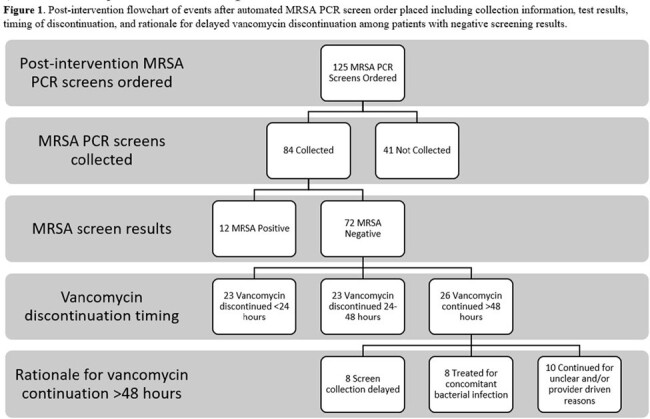

**Conclusion:**

Vancomycin treatment duration was shorter in patients after a pharmacy-based automated MRSA PCR screening intervention when implemented efficiently. This research will guide future interventions including pairing automated rapid testing with active stewardship response.

**Figure:**
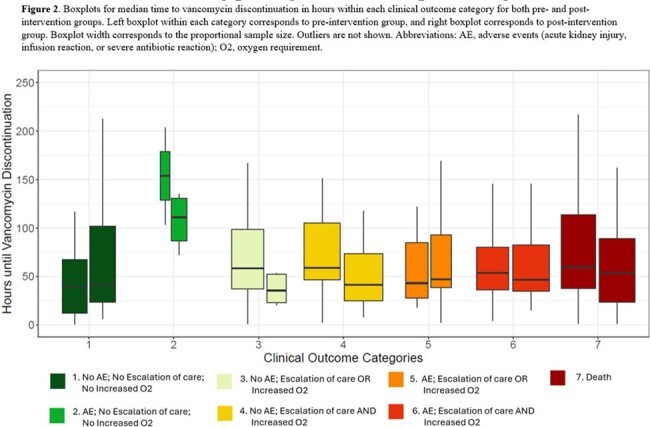

**Disclosures:**

**Elie Saade, MD, MPH, FIDSA**, Janssen Global Services: Advisor/Consultant|Janssen Global Services: Advisor/Consultant|Janssen Research and Development: Advisor/Consultant|Janssen Research and Development: Advisor/Consultant **Lisa M, Stempak, MD**, Cytovale Inc.: Advisor/Consultant

